# CT Combined with Multiparameter MRI in Differentiating Pathological Subtypes of Non-Small-Cell Lung Cancer before Surgery

**DOI:** 10.1155/2022/8207301

**Published:** 2022-05-17

**Authors:** Xinwen Li, Xiaoyan Wang, Qing Li, Lijie Bai

**Affiliations:** ^1^Department of CT Diagnosis, Yan'an People's Hospital, Yan'an, Shaanxi 716000, China; ^2^Department of Anesthesiology, The Affiliated Hospital of Yan'an University, Yan'an, Shaanxi 716000, China; ^3^Department of General Surgey Ward, Yulin First Hospital, Yulin, Shaanxi 719000, China

## Abstract

**Objective:**

To investigate the diagnostic value of computed tomography (CT) combined with multiparametric magnetic resonance imaging (mpMRI) for preoperative differentiation of non-small-cell lung cancer (NSCLC).

**Methods:**

CT and MRI imaging data were collected from all patients with squamous lung cancer and adenocarcinoma admitted to our hospital from June 2019 to December 2020 (286 cases). ROC curves were plotted to evaluate the performance of CT, mpMRI, and CT combined with mpMRI to differentiate pathological subtypes of NSCLC. Univariate and multivariate regression were used to be independent predictors of pathological subtypes of NSCLC.

**Results:**

ROC curves showed that CT combined with mpMRI had the largest area under the curve, followed by mpMRI and CT successively. Univariate regression analysis showed that gender, smoking, tumor size, morphology, marginal lobulation, marginal burr, bronchial truncation sign, and vascular convergence sign were factors influencing the pathological subtype of NSCLC. Multivariate regression analysis suggested the fact that gender, tumor size, morphology, marginal lobulation, bronchial truncation, and vascular convergence sign are likely the independent predictors of NSCLC pathological subtypes.

**Conclusions:**

CT combined with mpMRI can effectively distinguish NSCLC pathological subtypes, which is worthy of clinical application.

## 1. Introduction

Lung cancer, accounting for 11.6% of the total cancer cases, is the most common malignancy and the leading cause of cancer-related death worldwide [1]. Histopathologically, it can be divided into small-cell lung cancer (SCLC) and non-small-cell lung cancer (NSCLC), with the latter showing a higher frequency of 80% of all lung cancers [2]. NSCLC consists of three main histopathological types, namely, lung adenocarcinoma (LUAD), lung squamous cell carcinoma (LUSC), and large cell carcinoma [3], with LUAD (40–50%) and LUSC (20–30%) being the most common. LUAD and LUSC differ in terms of histology characteristics, anatomical location, and glucose metabolism, suggesting that they should also be targeted for optimal treatment strategies to improve clinical outcomes [4, 5].

At present, histological examination remains the dominant method for the diagnosis of lung cancer, which is mainly realized by surgery or lung biopsy [6]. However, surgery cannot be an option for some patients with underlying diseases, coagulation disorders, and poor lung function [7]. Lung biopsy is an invasive procedure that also entails surgical risks and potential complications [8]. It is difficult for lung biopsy to accurately characterize the spatial heterogeneity of the tumor because needle biopsy obtains a small sample of the tumor [9], and despite its high detection rate, invasive, time-consuming, laborious, and expensive factors diminish the advantages of lung biopsy. Serum markers also seem to distinguish NSCLC subtypes, such as tumor necrosis factor *α* (TNF-*α*), IL-6, IL-1*α*, IL-8, inflammatory chemokine CCL2, and CXCL12-CXCR4, which promote cell growth, survival, invasion, and angiogenesis. However, as of now, no effective models or specific indicators for early screening have been identified and remain to be developed.

In recent years, radiomics has been widely used in the early diagnosis, efficacy evaluation, and prognosis prediction of tumors and previous studies have shown that radiology has great clinical potential and is expected to be a new biomarker with satisfactory predictive performance [10]. Consequently, contrast-enhanced computed tomography (CT) and magnetic resonance imaging (MRI) of the lung are also increasingly used. CT has a high contrast resolution and can effectively detect lung cancer lesions, but the shortcoming is that CT has a high false positive rate for the detection of NSCLC [11]; although multiparametric MRI (mpMRI) is inferior to CT in terms of accuracy, mpMRI can characterize lung lesions because it has the best spatial resolution and soft tissue contrast and anatomical sequences (T1- and T2-weighted MRI) can clearly reflect the distribution of hilar and mediastinal lymph nodes [12–15]. Functional imaging sequences include diffusion-weighted MRI (DW-MRI) and dynamic contrast-enhanced MRI (DCE-MRI); the former has recently been noted to be beneficial for initial staging of lung cancer and assessment of lymph node status [16, 17]. Therefore, we believe that the combination of these imaging techniques can complement each other to some extent and has the potential to be a new assessment model for the early diagnosis of NSCLC subtypes. Accurate identification of pathological subtypes of NSCLC before surgery is of great clinical value in selecting treatment options and assessing prognosis. Therefore, in this study, we propose to use CT combined with mpMRI to distinguish LUAD and LUSC before treatment and to explore their clinical utility in depth, to provide new ideas and theoretical basis for clinical practice.

## 2. Materials and Methods

### 2.1. Study Subjects

The present study was a retrospective study, and informed consent was waived. 286 patients diagnosed with NSCLC treated in our hospital from June 2019 to December 2020 were selected as study subjects. All of them received preoperative CT and mpMRI and were confirmed to develop ADC or SqCC after pathological diagnosis. This study was approved by the Medical Ethics Committee of Yan'an People's Hospital (No. 20220001).

Inclusion criteria were as follows: (1) patients diagnosed with NSCLC; (2) patients without treatment before imaging examination; (3) patients with primary lung lesions; (4) patients with strong rectal peristalsis on mpMRI scan; (5) patients with contraindications to CT and MRI.

Exclusion criteria were as follows: (1) patients who were afflicted with other malignant tumors or combined tumors with distant metastasis; (2) patients with severe heart, lung, and other vital organ dysfunction; (3) patients who had received lung cancer treatment before imaging examination; (4) patients with strong rectal peristalsis in mpMRI scanning; (5) patients with contraindications of CT and MRI.

### 2.2. CT Scanning

Thin-section spiral DCE-CT scanning of the chest was performed (PHILIPS 256 iCT; TOSHIBA Aquilion ONE), with the following scanning parameters: slice thickness 5 mm, slice distance 5 mm; reconstruction slice thickness 1 mm, interval 1 mm, tube voltage 120 kV, and tube current 200 mA. Contrast-enhanced CT scanning was performed by injecting 100 ml of contrast agent iopromide (Vltravist-370, containing 370 g/L iodide) using a double-barrel high-pressure syringe at a flow rate of 3.5 mL/s. Dual-phase DCE-CT scanning (arterial and venous phases) was conducted at 25 s and 120 s after injection with the scanning ranging from the thoracic inlet to the adrenal glands.

### 2.3. mpMRI Scanning

One week within the discovery of pulmonary lesions in chest CT examination, all patients underwent conventional T2WI and EPI-DWI sequence scanning on the same 3.0 t MRI scanner (Achieva, Philips Healthcare, Best, Netherlands). The SENSE-XL-TORSO 8-channel phased array body coil and sensitivity encoding (SENSE) technology were used for the examination. Patients with a quiet and free breathing stage were scanned using end-expiratory triggered continuous scanning. MRI scanning included coronal T2WI, transverse T2WI, and EPI-DWI. The scanning range covered the thoracic inlet to the lower border of the diaphragm. Images were exported in the DICOM format. Specific scanning parameters included the following: transverse T2WI: T2WI/TSE, TR/TE (ms) = 992/80, NSA = 1, FOV = 430 mm × 350 mm, matrix 360 × 249, slice thickness/interslice gap (mm) = 5.0/0.5, and scanning time = 23 seconds; transverse EPI-DWI: TR/TE (ms) = 1306/54, NSA = 3, FOV = 430 mm × 336 mm, matrix 144 × 109, slice thickness/interslice gap (mm) = 5.0/0.5, scanning time = 1.05 min, and *b* value = 0, 20, 40, 200, and 800 s/mm^2^.

### 2.4. Assessment of Model Effectiveness

The area under the curve (AUC) of the ROC curve was used to evaluate the diagnostic efficacy of the imaging examinations in predicting NSCLC pathological subtypes before surgery. Their accuracy, sensitivity, and specificity were calculated. Furthermore, univariate and multivariate regressions were adopted to analyze the independent predictors of NSCLC pathological subtypes.

### 2.5. Statistical Analysis

The statistical software package used was SPSS 22.0. Enumeration data were represented as *n* (%), and the chi-square test was used for comparing the results between groups. Logistic regression was used to analyze the influencing factors of NSCLC pathological subtypes; the ROC curve played a role in evaluating the diagnostic value of each detection method. The difference was statistically significant when *P* < 0.05.

## 3. Results

### 3.1. Clinical Features of Patients

As shown in Table 1, significant statistical differences were observed between LUAD and LUSC patients in gender, smoking, tumor size, tumor morphology, marginal lobulation, marginal burr, bronchial truncation, and vascular convergence sign. Compared with LUAD patients, firstly, LUSC patients had a higher proportion of males, smokers, and mass-like lesions and had larger tumor size (all *P* < 0.05). In addition, in patients with LUSC, there were more marginal lobulation and fewer marginal burrs of the mass and less bronchial truncation sign and vascular convergence sign (all *P* < 0.05). However, there were no significant statistical differences in age, tumor location, tumor stage, histological differentiation, and lymph node metastasis between LUAD and LUSC patients (all *P* > 0.05).

### 3.2. CT Scanning, mpMRI, and CT Combined with mpMRI in Distinguishing NSCLC Pathological Subtypes

Criteria for positive/negative results were as follows: true positive demonstrated that imaging examination and pathological diagnosis suggested LUAD; false positive demonstrated that imaging examination showed LUAD but pathological diagnosis indicated LUSC; false negative demonstrated that imaging examination showed LUSC but pathological diagnosis suggested LUAD; true negative demonstrated that both the imaging examination and pathological diagnosis indicated LUSC.  Table 2 lists the results of CT, mpMRI, and CT combined with mpMRI in differentiating between LUAD and LUSC. Compared with using CT scan or mpMRI alone, CT combined with mpMRI performed better in diagnosing pathological subtypes of NSCLC, with 121 true positive cases and 101 true negative cases.

### 3.3. ROC Curve in Assessing the Performance of CT, mpMRI, and CT Combined with mpMRI in Distinguishing NSCLC Pathological Subtypes

**Figure 1:**
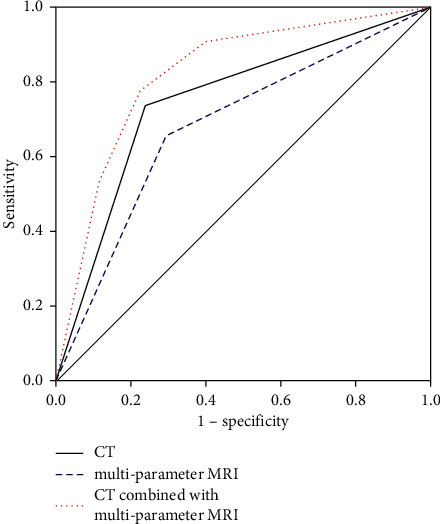
ROC curve.

The results showed that the AUC (0.749), sensitivity (73.7%), specificity (76.2%), positive predictive value (78.8%), negative predictive value (70.7%), and accuracy (74.8%) of CT were higher than those of mpMRI (0.681, 65.4%, 70.8%, 72.9%, 63.0%, and 67.8%). Also, when CT was combined with mpMRI, all the indicators were higher than those of CT or mpMRI alone (0.824, 77.6%, 77.7%, 80.7%, 74.3%, and 77.6%). This result suggests that CT + mpMRI has a high accuracy rate in differentiating pathological subtypes of NSCLC (Table 3 and Figure 1)

### 3.4. Univariate and Multivariate Regression Analysis of Independent Predictors of NSCLC Pathological Subtypes

In addition, we used regression analysis of clinical characteristics as independent predictors of pathological subtypes of NSCLC (Table 4). Univariate analysis showed that gender, smoking, tumor size, morphology, marginal lobulation, marginal burr, bronchial truncation sign, and vascular convergence sign could be independent predictors of pathological subtypes of NSCLC. Furthermore, the results of the multifactorial analysis showed that gender (*P*=0.004; OR, 0.463; 95% CI: (0.273–0785)), tumor size (*P*=0.004; OR, 2.299; 95% CI: (1.313–4.027)), tumor morphology (*P*=0.006; OR, 2.157; 95% CI: (1.241–3.751)), marginal lobulation (*P*=0.004; OR, 2.264; 95% CI: (1.299–3.946)), bronchial truncation sign (*P*=0.004; OR, 2.223; 95% CI: (1.296–3.813)), and vascular convergence sign (*P*=0.003; OR, 0.441; 95% CI: (0.259–0.751)) could predict the subtypes of NSCLC. Patients who were male, had larger tumors, had more marginal lobulation, had bronchial truncation signs, and had fewer vascular convergence signs had a higher probability of belonging to LUSC.

## 4. Discussion

NSCLC remains the chief culprit of global cancer-caused mortality. Despite recent advances in this field, the five-year survival rate of lung cancer patients has not improved significantly, which is mainly due to unsatisfactory detection and treatment strategies [17]. Histological subtypes of NSCLC, mainly including LUAD, LUSC, and large cell carcinoma, share multiple common biological features, but they vary from one another in terms of original cell, location in the lung, and growth pattern, suggesting that they are different diseases with different molecular mechanisms [2].

Furthermore, it has been found that different subtypes manifest different patterns of genetic alterations [18, 19]. Existing clinical trials also suggested that tumor subtypes affected response rate, toxicity, and progression-free survival of targeted agents such as bevacizumab, pemetrexed, and epidermal growth factor receptor tyrosine kinase inhibitors (EGFR-TKIs) [20, 21]. Hence, the accurate categorization of histological subtypes can contribute to developing better targeted therapeutic strategies in clinical practice. In addition, differentiation of subtypes will promote the progress of the study and implementation of targeted therapy for lung cancer, so as to offer more adaptive diagnosis and treatment plan for patients and ultimately to prolong patient survival as much as possible.

Clinically, there are many challenges confronting the traditional diagnosis of histological subtypes of lung cancer, which mainly relies on postoperative invasive biopsy and pathological tissue sections. However, surgery with uncertainty will cause damage to the patient's body and lesion area. Furthermore, this detection has time effect and may delay the diagnosis; the lesion cannot be monitored frequently during a short period, and the pathological tissue section can only be prepared after surgery. CT and MRI are currently the main noninvasive imaging methods for the diagnosis of NSCLC lesions. The former, with high temporal, spatial, and contrast resolution, can show the size, morphology, and location of any lung tumor and is able to detect unfiltered lymph nodes with cancer cells [22]. Nevertheless, CT has ionizing radiation and a low detection rate of small lesions. Some patients are allergic to the iodine contrast medium and are thus unsuitable for contrast-enhanced CT scanning. Chest MRI has also long been technically challenging due to motion and respiratory artefacts of the thoracic organs as well as susceptibility artefacts caused by interfaces between different tissues and the overall low proton density of the lung [23]. With the continuous advancement of MRI technology, it has been suggested that MRI, DW-MRI in particular, can be a good alternative to CT [12, 24]. DCE-MRI can present the blood supply characteristics of lesions and obtain tumor perfusion signal, which contributes greatly to the detection of the lesion [13]. However, in MRI examination, some cases are unable to hold breath for a relative long time, resulting in respiratory artifacts and affecting the observation of the focus [25]. Besides, a very small number of patients cannot complete an MRI examination due to claustrophobic syndrome or ferromagnetic objects in the body that cannot be removed. At present, it has been stated that DW-MRI plays an equivalent role to CT in detecting tumor volume in NSCLC patients after chemotherapy and can even reliably determine the range of radiation-induced lung toxicity [26]. Gkogkozotou et al. [22] have found that the combination of PET/CT and brain MRI is beneficial to accurately classifying the stages of NSCLC and reduce avoidable thoracotomy. One study has discovered that the CT-based radiomics model can predict malignancy in pulmonary nodules (<1 cm) [27]. However, in clinical application, missed diagnosis and misdiagnosis can happen when CT or MRI is applied alone on account of their limitations. Having discussed all the above content, we may arrive at the conclusion that it is of great clinical value to combine CT with mpMRI in the detection of NSCLC lesions.

In this study, a thorough assessment of CT, mpMRI, and CT combined with mpMRI in differentiating the ADC and SqCC subtypes of the 286 included NSCLC patients was carried out. The findings showed that mpMRI with CT could accurately detect NSCLC lesions with a higher detection rate than that of CT or MRI alone. In conclusion, CT and MRI have their own advantages and limitations, and the combination of them can complement each other fairly; MRI with high sensitivity and accuracy can be applied to the preoperative routine examination of NSCLC, and CT scanning can act as an adjuvant.

This study still has the following limitations: First, this was a single-center study with a small sample size, which may affect the credibility of the results. Hence, more multicenter studies with large sample sizes are required. In addition, this study mainly focused on LUAD and LUSC and thus failed to cover all NSCLC cases and failed to meet the needs of clinical application. This deficiency may also affect the credibility of the results.

In conclusion, we propose the application of CT combined with mpMRI in distinguishing preoperative NSCLC pathological subtypes. According to the results, such combined detection has a favorable complementary effect and can increase the diagnosis accuracy. In future studies, we shall focus on imaging methods for accurate diagnosis and classification of all subtypes of NSCLC.

## Figures and Tables

**Table 1:** Comparison of indicators between the two groups.

Features	Total cases	LUAD (*n* = 130)	LUSC (*n* = 156)	*X* ^2^	*P*
*Gender (%)*					
Male	167 (58.4)	60 (46.2)	107 (68.6)	14.691	≤0.001
Female	119 (41.6)	70 (53.8)	49 (31.4)		
*Age (%)*					
<65 year old	87 (30.4)	43 (33.1)	44 (28.2)	0.795	0.373
≥65 year old	199 (69.6)	87 (66.9)	112 (71.8)		
*Smoking (n, %)*					
No	112 (39.2)	60 (46.2)	52 (33.3)	4.892	0.027
Yes	174 (60.8)	70 (53.8)	104 (66.7)		
*Location (%)*					
Superior lobe of the left lung	57 (19.9)	29 (22.3)	28 (17.9)	4.146	0.387
Inferior lobe of the left lung	58 (20.3)	20 (15.4)	38 (24.4)		
Superior lobe of the right lung	64 (22.4)	30 (23.1)	34 (21.8)		
Middle lobe of the right lung	60 (21.0)	27 (20.8)	33 (21.2)		
Inferior lobe of the right lung	47 (16.4)	24 (18.5)	23 (14.7)		
*Tumor size (%)*					
<3 cm	183 (64.0)	94 (72.3)	89 (57.1)	7.162	0.007
≥3 cm	103 (36.0)	36 (27.7)	67 (42.9)		
*Tumor staging (%)*					
Stages I-II	160 (55.9)	77 (59.2)	83 (53.2)	1.045	0.307
Stages III-IV	126 (44.1)	53 (40.8)	73 (46.8)		
*Tissue differentiation degree (%)*					
Low and middle	186 (65.0)	79 (60.8)	107 (68.6)	1.907	0.167
High	100 (35.0)	51 (39.2)	49 (31.4)		
*Lymphatic metastasis (%)*					
No	220 (76.9)	105 (80.8)	115 (73.7)	1.986	0.159
Yes	66 (23.1)	25 (19.2)	41 (26.3)		
*Tumor morphology (%)*					
Nodule	180 (62.9)	94 (72.3)	86 (55.1)	8.972	0.003
Mass	106 (37.1)	36 (27.7)	70 (44.9)		
*Marginal lobulation (%)*					
No	100 (35.0)	60 (46.2)	40 (25.6)	13.121	≤0.001
Yes	186 (65.0)	70 (53.8)	116 (74.4)		
*Marginal burrs (%)*					
No	138 (48.3)	54 (41.5)	84 (53.8)	4.302	0.038
Yes	148 (51.7)	76 (58.5)	72 (46.2)		
*Bronchial truncation sign (%)*					
No	112 (39.2)	63 (48.5)	49 (31.4)	8.653	0.003
Yes	174 (60.8)	67 (51.5)	107 (68.6)		
*Vascular convergence sign (%)*					
No	156 (54.5)	59 (45.4)	97 (62.2)	8.067	0.005
Yes	130 (45.5)	71 (54.6)	59 (37.8)		

*Note.* LUAD, lung adenocarcinoma; LUSC, lung squamous cell carcinoma.

**Table 2:** Diagnosis of CT scanning, multiparameter MRI (mpMRI), and CT combined with mpMRI.

Pathological diagnosis	CT		mpMRI		CT combined with mpMRI	
	LUSC	LUAD	LUSC	LUAD	LUSC	LUAD
LUSC	115	41	102	54	121	35
LUAD	31	99	38	92	29	101

*Note.* CT, computed tomography; mpMRI, multiparameter magnetic resonance imaging.

**Table 3:** Assessment of the diagnostic value of the three detection methods.

	AUC	Sensitivity (%)	Specificity (%)	Positive predictive value (%)	Positive predictive value (%)	Accuracy (%)
CT	0.749 (0.691–0.808)	73.7 (66.8–80.6)	76.2 (68.8–83.5)	78.8 (72.1–85.4)	70.7 (63.2–78.3)	74.8 (69.8–79.9)
mpMRI	0.681 (0.618–0.743)	65.4 (57.9–72.9)	70.8 (63.0–78.6)	72.9 (65.5–80.2)	63.0 (55.2–70.8)	67.8 (62.4–73.2)
CT combined with mpMRI	0.824 (0.774–0.874)	77.6 (71.0–84.1)	77.7 (70.5–84.8)	80.7 (74.3–87.0)	74.3 (66.9–81.6)	77.6 (72.8–82.5)

*Note.* CT, computed tomography; mpMRI, multiparameter magnetic resonance imaging.

**Table 4:** Univariate and multivariate regression analyses.

Features	Univariate analysis		Multivariate analysis	
	OR (95% CI)	*P*	OR (95% CI)	*P*
Gender	0.393 (0.242–0.636)	≤0.001	0.463 (0.273–0.785)	0.004
Age	1.258 (0.759–2.085)	0.373		
Smoking	1.714 (1.062–2.768)	0.028	1.499 (0.877–2.561)	0.139
Location				
Superior lobe of the left lung	1.000			
Inferior lobe of the left lung	1.968 (0.929–4.167)	0.077		
Superior lobe of the right lung	1.174 (0.574–2.399)	0.660		
Middle lobe of the right lung	1.266 (0.612–2.619)	0.525		
Inferior lobe of the right lung	0.993 (0.458–2.149)	0.985		
Tumor size	1.966 (1.195–3.235)	0.008	2.299 (1.313–4.027)	0.004
Tumor staging	1.278 (0.798–2.045)	0.307		
Tissue differentiation degree	0.709 (0.435–1.156)	0.168		
Lymphatic metastasis	1.497 (0.852–2.630)	0.160		
Tumor morphology	2.125 (1.293–3.494)	0.003	2.157 (1.241–3.751)	0.006
Marginal lobulation	2.486 (1.511–4.090)	≤0.001	2.264 (1.299–3.946)	0.004
Marginal burr	0.609 (0.381–0.974)	0.039	0.651 (0.384–1.104)	0.111
Bronchial truncation	2.053 (1.268–3.326)	0.003	2.223 (1.296–3.813)	0.004
Vascular convergence	0.505 (0.315–0.811)	0.005	0.441 (0.259–0.751)	0.003

## Data Availability

The data used to support the findings of this study are available from the corresponding author upon request.
